# Plasma lactate measurement as an example of encountered gaps between routine clinical laboratory processes and manufactures' sample-handling instructions

**DOI:** 10.1016/j.plabm.2018.e00109

**Published:** 2018-10-24

**Authors:** Ibrahim A. Hashim, Mishkat Mohamed, Aileen Cox, Fernabelle Fernandez, Patricia Kutscher

**Affiliations:** a Clinical Laboratories, Parkland Health and Hospital System, United States; b Department of Pathology, University of Texas Southwestern Medical Center, Dallas, TX, USA

**Keywords:** Lactate, Sample transportation, Sepsis, Pre-analytical factors

## Abstract

**Objectives:**

Deviation from manufacturers’ pre-analytical sample handling recommendations necessitates extensive validation studies. This report uses plasma lactate testing, where a recommended 15 min room temperature sample handling limit cannot be met by the clinical laboratory, as an example for studies to bridge the gap with practice.

**Design and Methods:**

Triplicate blood samples were collected from patients (n = 51) with lactate requests by clinicians and from normal volunteers (n = 50). One tube was transported on ice (4 °C), the others were maintained at room temperature (23 °C). Tubes stored at 4 °C were processed at 30 min from collection. Tubes stored at 23 °C were processed at 15 and at 30 min from collection. Lactate levels were measured using Roche Diagnostics Cobas 6000® analyzer.

**Results:**

Lactate levels in normal subjects ranged from 0.6 to 3.1 mmol/L (median 1.1). Patient lactate levels ranged from 0.8 to 26.3 mmol/L (median 2.2). Bias in lactate levels following extended storage of samples from both normal subjects and patients ranged from − 1.3 to2.2 and from − 1.0–1.0 mmol/L when stored for 30 min at 23 °C or at 4 °C, respectively. The bias between lactate levels at 30 min at 23 °C and 4 °C was − 1.2 to − 0.5 mmol/L for both populations. Although the bias was not statistically significant for all variables, a clinically significant (>0.2 mmol/L) bias was observed in 28% of normal and 7.0% of patient samples.

**Conclusion:**

Extending the pre-analytical time to 30 min at 23 °C did not significantly impact clinical utility of lactate measurement in our patient population.

## Highlights

• Pre-analytical gaps exist between manufacturers’ recommendation and clinical practice.
• Sample handling time for lactate testing was extended to reflect clinical practice.
• Normal subjects, often preferred, showed high variability compared to patients.
• Patient samples must always be included in pre-analytics studies.
• Doubling handling time at 23 °C did not have significant clinical impact.

## 1 Introduction

Clinical laboratories are required to adhere to manufacturers’ recommended sample handling instructions as part of their compliance with accreditation certification [1]. However, manufacturer's recommendations do not always reflect current practice and deviations from recommendations necessitate extensive validation studies [2]. This report uses plasma lactate testing, integral to assessment of tissue perfusion, metabolic derangement, and to sepsis risk assessment protocols [3], [4], as an example.

Lactate is an intermediary metabolite produced in relatively anaerobic conditions. Measurement of circulating lactate level is useful in assessing tissue perfusion in critically ill patients and in those being suspected of sepsis. It is produced by many cells and circulating levels reflect rate of production and of metabolism by the body.

Lactate measurements are performed using either laboratory-based automated analyzers and or point-of-care devices including those offering blood gas analysis. Blood samples sent for measurement using distant laboratory-based instruments are often collected in tubes containing sodium fluoride-potassium oxalate as a preservative to reduce glycolysis and thus in vitro lactate production. Recommendations for sample handling among instrument manufacturers are varied for both storage and for transportation conditions and time allowed for analysis from collection. Some manufacturers recommend 15–30 min at room temperature (23 °C) whereas others recommend placement on ice (4 °C) [6], [7], [8].

Our reagent manufacturer recommends that samples for lactate testing be transported without ice at ambient temperature and be analyzed within 15 min of collection [6]. At our institution samples transit may take up to 30 min and that samples delayed beyond 15 min are rejected and a re-collection is requested. This causes delay in patient care, unnecessary blood loss and patient and clinician dissatisfaction.

Several studies have examined sample stability for lactate measurements [9], [10], [11], [12], however, in most this was performed primarily in samples from normal subjects. To our knowledge, this is the only study that examined handling conditions for lactate in the setting of routine clinical practice for patients being investigated for sepsis and or metabolic and hemodynamic disorders. This study examined the effect of extending sample transit time to 30 min at either 23 °C or at 4 °C to reduce the number of samples rejections.

## 2 Materials and methods

2.1. Triplicate blood samples were collected from patients (n = 51) (with lactate requested by physicians as part of their clinical care) and from normal volunteers (n = 50).
2.2. One blood tube was placed on crushed ice (4 °C), the other two tubes were maintained at room temperature (23 °C). All tubes were transported to the clinical laboratory within 10 min of collection.
2.3. For blood tubes stored at 23 °C, one was processed at 15 min and the other at 30 min from collection. Tubes stored at 4 °C were processed at 30 min from collection.
2.4. Lactate was measured by an enzymatic lactate oxidase assay using the Cobas 6000® analyzer, (Roche Diagnostics, IN, USA) [6].
2.5. Bias in lactate levels obtained on samples stored at 23 °C and at 4 °C for 30 min was calculated as compared with those obtained at the recommended 15 min at 23 °C.
2.6. Manufacturers’ published reference intervals were re-validated using samples from normal volunteers. Samples were subjected to all of the above sample handling variables.
2.7. Demographics (age and gender) of study subjects were recorded.
2.8. Patients clinical findings and diagnosis as well as associated blood gases results were recorded to assess diagnostic sensitivity and specificity.
2.9. Statistical analysis for ANOVA, non-parametric Mann-Whitney test, tests for normality, and correlations were performed using Microsoft Excel® software (Microsoft, Inc., CA, USA) and Minitab®17 (Minitab, PA, USA).

## 3 Results

A total of 123 samples were obtained from patients (n = 51) (23 females, 28 males) with age ranging from 23 to 83 (mean 53) years, being investigated for sepsis, hemodynamic and associated metabolic abnormalities and from normal volunteers (n = 50) (37 females, 13 males) with age ranging from 31 to 59 (mean 50) years. Patients’ diagnosis and clinical findings are shown in Table 1.

**Table 1:** Patients diagnosis and clinical findings. All of the encountered clinical scenarios above can lead to lactic acidosis as a consequence of hypoxia, infection, increased metabolism, reduced tissue perfusion, reduced lactate metabolism and clearance.

Diagnosis / Clinical findings	Number of patients
Sepsis and septic shock	15
Acidosis (Unknown etiology)	6
Malignancies (Lung, gastrointestinal, bladder, Leukemia)	6
Alcoholic Cirrhosis	5
Trauma	5
Heart Failure	4
Renal Disorder	4
Respiratory failure	3
Pancreatitis	2
Abdominal pain	1

**Fig. 1:**
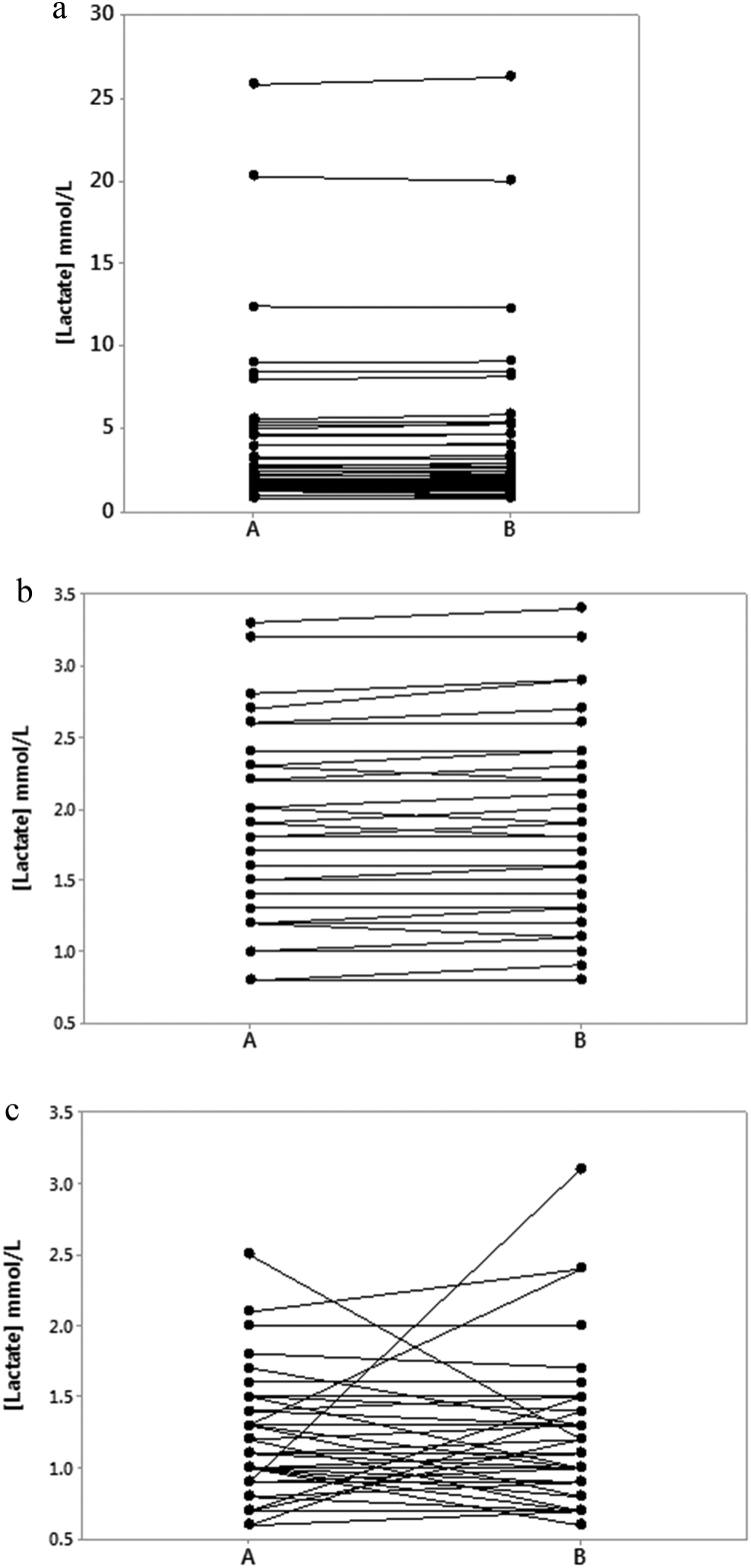
a: Lactate levels in samples from patients when subjected to the manufacturer's recommended pre-analytical conditions (A) 15 min at 23 °C and for the experimental condition (B) of 30 min at 23 °C. b: Lactate levels in samples from patients when subjected to the manufacturer's recommended pre-analytical conditions (A) 15 min at 23 °C and for the experimental condition (B) of 30 min at 23 °C. Narrow view showing lack of variable response when lactate concentrations are< 3.5 mmol/L similar to those observed in normal subjects (Fig. 1c). c: lactate levels in samples from normal individuals when subjected to the manufacturer's recommended pre-analytical conditions (A) 15 min at 23 °C and for the experimental condition (B) of 30 min at 23 °C.

Lactate levels among normal subjects ranged from 0.6 to 3.1 mmol/L (median 1.1). Patients’ lactate levels ranged from 0.8 to 26.3 mmol/L (median 2.2) and were significantly elevated when compared with lactate in normal subjects (P < 0.005). The range and median levels observed in normal subjects and in the patient population for each of the three pre-analytical sample handling conditions (15 and 30 min at 23 °C) and (30 min at 4 °C) are all shown in Table 2. Although extending sample storage showed variable lactate response in normal subjects when compared to patients, this was not statistically significant (P = 0.63) (Fig. 1a–c).

**Table 2:** Range and median lactate levels in patients and in normal subjects when processed at the recommended pre-analytical sample handling conditions (15 min at 23 °C), and at the experimental 30 min at 23 °C and 30 min at 4 °C following collection. When compared with manufacturer recommended condition (15 min at 23 °C), there was no significant difference, using analysis of variance (ANOVA, Minitab software) between patient or normal subjects lactate levels obtained for the different study variables.

Study subjects	Lactate levels (range and (median)) mmol/L		
	Pre-analytical sample transit time and storage conditions		
	15 min at 23 °C	30 min at 23 °C	30 min at 4 °C
Patients	0.8–25.5 (2.2)	0.6–26.3 (2.2)	0.8–26.1 (2.1)
Normal subjects	0.6–2.5 (1.1)	0.6–3.1 (1.1)	0.6–2.0 (1.1)

In normal subjects, lactate levels in samples transported and stored for an extended 30 min at 23 °C were not significantly different from those processed as recommended (15 min at 23 °C) (P = 0.68), similarly for samples transported and stored for 30 min at 4 °C (P = 0.59). Samples transported and stored for 30 min at 23 °C were not significantly different from those stored and transported for the same time at 4 °C (P = 0.37) (Table 2).

Lactate levels in patient samples transported and stored for 30 min at 23 °C were not significantly different from those either processed as recommended (15 min at 23 °C) (P = 0.99) or, transported and stored for 30 min at 4 °C (P = 0.98) (Table 2).

There were strong correlations between patient lactate levels obtained for the different pre-analytical variables (r > 0.99, (P < 0.005)) for all, whereas, in normal subjects, correlations (r^2^ values) between lactate levels obtained for the different pre-analytical variables were 0.44, 0.64 and 0.92 (P < 0.005 for all) for 15 and 30 min at 23 °C, for 15 and 30 min at 23 °C and, for 30 min at 23 °C and 4 °C, respectively.

As part of their clinical management, 37 patients (73%) had blood gases requests associated with requests for lactate. In those patients, arterial blood pH levels ranged from 7.17 to 7.53 (mean 7.42), pCO2 levels ranged from 16 to 49 (mean 32), and bicarbonate (HCO_3_) levels ranged from 6 to 29 (mean 19) mmol/L. There was poor but significant negative correlation between blood gases results and lactate levels (r < −0.5) (pH (P = 0.12), pCO_2_ (P < 0.003), and HCO_3_ (P < 0.005)).

**Fig. 2:**
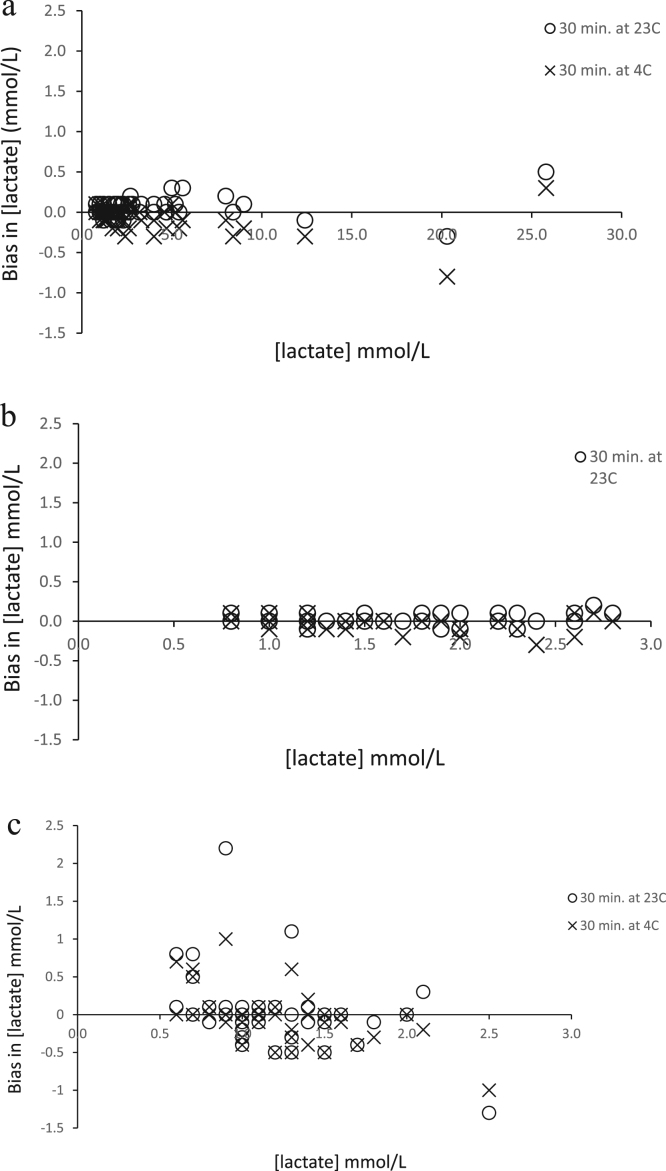
a: Bias in lactate levels among samples from patients when handled as per manufacturer's recommendation (15 min at 23 °C) and the experimental pre-analytical conditions of; 30 min at 23 °C (О) and at 4 °C (✕). b: Narrowed view (up to 3.5 mmol/L range similar to range in normal subjects (Fig. 2c) showing bias in lactate concentration among patient samples when handled as per manufacturer's recommendation (15 min at 23 °C) and the experimental pre-analytical conditions of; 30 min at 23 °C (О) and at 4 °C (✕). c: Bias in lactate levels among samples from normal individuals when handled as per manufacturer's recommendation (15 min at 23 °C) and the experimental pre-analytical conditions of; 30 min at 23 °C (О) and at 4 °C (✕).

In samples from normal individuals, bias in lactate levels ranged from − 1.3–2.2 mmol/L (median 0) when stored at 23 °C for 30 min, and from − 1.0–1.0 mmol/L (median 0) when stored at 4 °C for 30 min when compared with recommended pre-analytical conditions (15 min at 23 °C). Bias in lactate levels between samples stored at 23 °C and at 4 °C for 30 min ranged from –1.2–0.3 (median 0) mmol/L (Fig. 2c).

Similarly, for patients’ samples, bias in lactate levels ranged from − 0.3 to 0.5 mmol/L (median 0) when stored at 23 °C for 30 min and from −0.8to 0.3 (median 0) mmol/L when stored at 4 °C for 30 min. Bias in lactate levels when stored at 23 °C and at 4 °C for 30 min ranged from 0.1 to −0.5 mmol/L (median −0.1) (Fig. 2a, b).

Among samples from normal individuals, 14 samples (28%) exhibited a lactate bias> 0.2 mmol/L whereas only 3 (6%) showed a bias of> 0.8 mmol/L when stored for 30 min at 23 °C. Additionally, when stored for 30 min at 4 °C, 15 samples (30%) showed a bias> 0.2 and 2 (4%) showed a bias> 0.8 mmol/L. In contrast for patient samples, where only 4 samples (7.8%) exhibited a bias of> 0.2 mmol/L and none (0.0%) had a bias> 0.8 mmol/L with extended storage for 30 min at 23 °C. When stored for 30 min at 4 °C, 6 samples (12%) exhibited a bias> 0.2 mmol/L and none (0%) were> 0.8 mmol/L.

Among patients (n = 10) with significant bias in lactate levels considered to be significant (>0.2 mmol/L), 3 patients had septic shock, 2 with renal dysfunction, 3 with malignancy, and 3 with circulatory failure.

Inter-assay imprecision over 10 days for aliquots from 20 patients with mean lactate values of ranged from 0.0% to 6.7% (for 15 min at 23 °C), 0–13.1% (for 30 min at 23 °C) and 0–10.2% (for 30 at 4 °C) for samples with mean lactate values of 1.3, 5.5, 9.0, and 12.4 mmol/L respectively.

**Fig. 3:**
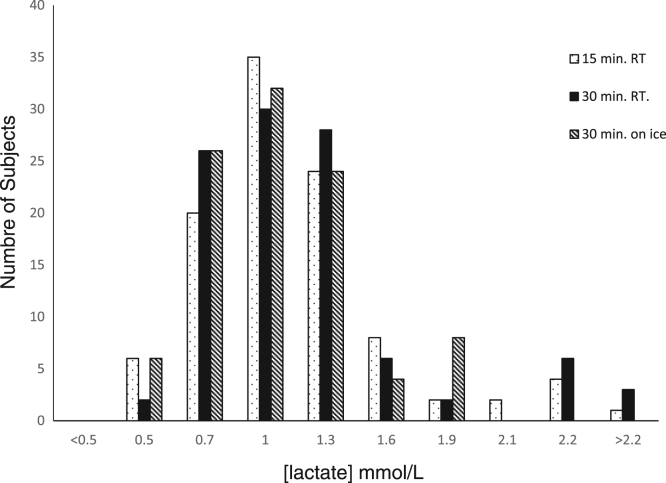
Lactate levels distribution in normal subjects within the recommended sample handling conditions (15 min at 23 °C) with reported reference intervals of 0.5–2.2 mml/L, and the distribution in lactate levels when the pre-analytical transit and storage conditions were changed to 30 min at 23 °C and for 30 min at 4 °C. More than 95% of samples (47 samples) for each of the different pre-analytical study variables were within the reported reference intervals.

The manufacturer's published reference intervals for lactate (0.5–2.2 mmol/L) was verified using samples from normal individuals subjected to the three different pre-analytical variables (Fig. 3)

## 4 Discussion

This study examined the impact of extending transit time for lactate measurement from that recommended by the manufacturer. Although several studies [9], [10], [11] have shown increased lactate levels due to in-vitro production, those studies were mainly conducted using samples from normal subjects. This study evaluated the impact on samples from both normal subjects and from patients presenting to the emergency department and being investigated for sepsis and or for metabolic and hemodynamic disorders.

Blood samples left standing on cells leads to elevation of intermediary metabolites such as lactate and ammonia due to continued cellular metabolism and removal from cells by centrifugation limits in-vitro production. Furthermore, incubation on ice reduces metabolism and thus rate of conversion of pyruvate to lactate. A well-preserved sample will provide a lactate level closely resembling circulating levels. Some of the limitations although valid, remains difficult to avoid as with a similar scenario we previously reported for ammonia [13]

Test reagent manufacturers require that blood specimens be processed within 15 min from collection. This transit time limit, however, cannot always be met particularly when the laboratory is distant from patient care areas, as the case in our institution where 3% of lactate samples arrive late into the laboratory and are thus rejected and a repeat collection is requested.

This causes delay in care, patient discomfort as well as patient and clinical care provider dissatisfaction. Equally important, repeated blood collection increases the risk for iatrogenic anaemia.

In an attempt to reduce the number of rejected lactate specimens and to bridge the pre-analytical gap with routine practice, this study examined the impact of increasing the pre-analytical time, from the recommended 15 min by our assay manufacturer to 30 min while sample transport and storage maintained at either room temperature (23 °C) or at 4 °C on ice.

It has been suggested [11] that an increase in lactate levels greater than 0.2 mmol/L is clinically significant. Furthermore, when using anti-retroviral drugs, an increase of 0.3–0.8 mmol/L was considered significant [10]. In our study, in-vitro lactate production was less than 0.8 mmol/L among patient samples and up to 1.2 mmol/L in samples from normal subjects. Although the bias was not statistically significant for all variables, a clinically significant (>0.2 mmol/L) bias was observed in 28% of normal and 7.0% of patient samples.

The bias was lower when samples were placed on ice (at 4 °C) and that the difference was not significant among patient population. Interestingly, this significance in bias was only apparent among normal subjects as described above.

Monitoring lactate levels is used as a target for directing therapy where decreasing lactate by 20% every 2 h for 8 h is thought to reduce ICU length of stay as well as mortality [5]

Samples obtained from the study patients and subjected to the same pre-analytical variables did not exhibit marked bias, even within the normal lactate ranges. The etiology of this is unclear, however, it may reflect reduced metabolism (sick cell syndrome), presence of inhibitors, or to pyruvate depletion in the patients, where by nature of the request they are often critically ill. This does not seem to be related to lactate levels as those from patients with lactate levels within the normal limits did not exhibit the degree of variability seen in the study patients. Therefore, since the performance of the assay for samples kept for 30 min either at 23 °C or 4 °C was not significantly different and that access to crushed ice may lead to additional sample delay and carries the risk of leakage during pneumatic tube transport, it was decided that samples transported at room temperature would be preferred. Our institution changed the lactate processing protocol to 30 min, leading to a reduction of lactate order rejections from 3% to less than 1%.

One important additional consideration when examining changes in sample handling, is the impact on reported reference intervals. Analysis of data obtained from the normal subjects group showed that the current reference intervals 0.5–2.2 mmol/L were transferable for the extended sample pre-analytical time. The study also provided validation for manufacturer reported reference intervals under the various pre-analytical variables not previously reported.

It is important that falsely elevated lactate levels are avoided as they may lead to additional unnecessary invasive or expensive investigation. Similarly, in settings where excessive sample transit time is a limitation, unexplained high lactate level may be attributed to poor sample quality.

Several studies have reported impact by pre-analytical factors. One reported that extended application of tourniquet from the preferred 2–8 min resulted in an increase of 0.1–0.2 mmol/L lactate [14]. One study, in addition to normal subjects, examined the effect on HIV patients receiving anti-retroviral medication known to impair lactate metabolism [11]. Another study looked at much extended sample storage up to 360 min, much beyond clinical relevance for intervention, but might be helpful in research setting or retrospective reviews based on blood from four normal subjects [10]. In one study, significant differences in lactate levels were seen when samples were stored at room temperature for up to 30 min by emergency medical services in an outpatient setting, however, lactate levels were stable when the samples were stored on ice for the same time period [11], similar findings to our study.

Using samples from study rodents, one study showed the need to immediately separate the sample from cells to avoid in-vitro lactate production [9]. Separating plasma from cells at bedside or at nursing stations is not feasible, furthermore, it would require aliquoting of samples prior to pneumatic tube transportation, all of those technical processes are best handled by technical staff and not nursing or medical staff.

Although there were no statistically significant differences between the outcomes of the various study variables for both population, the bias in lactate levels was more apparent in samples from normal subjects. However, the bias was not apparent in samples from patient population. 28% of normal subjects had a clinically significant bias compared with only 7.8% of the patient population. This supports our recommendation that patient population samples need to be included when examining pre-analytical variability on assay utility.

This study examined the impact on lactate measurement in real patient care scenarios for the extended time that represents the gap with manufacturers recommended practice. It is important to note that one manufacturer recommends removal of plasma from cells as soon as possible after collection (no time limit indicated) and immediate analysis [8]; another manufacturer recommends sample be transported on ice, separated from cells within 30 min, and be analyzed immediately after centrifugation [7]. Most manufacturers recommend use of sample anticoagulants with lithium heparin being most popular as it facilitates faster analysis of plasma to avoid waiting on sample clotting for serum. However, others recommend fluoride-oxalate either as sole tube or as available option among other tubes. A mixture of 10 mg of sodium fluoride and 2 mg of potassium oxalate per ml of blood has long been shown to completely inhibits glycolysis for 8 h when combined with storage at 4 °C [15]

Increase in circulating lactate levels can occur in a number of conditions and not singularly attributed to hypoxia. For instance, liver dysfunction (decreased metabolic clearance), diabetes, haematological malignancies, drugs such as alcohols, and adrenergic agonist, in-born errors of metabolism, and hypoglycemia [18]. Among our study patient population, 47% had lactate levels above 2.2 mmol/L. Although 28.8% of patients had either sepsis or were in septic shock, several had hepatic cirrhosis secondary to ethanol abuse, cardiac failure, renal impairment, solid and haematological malignancies known to be associated lactic acidosis [16], [17], as well as metabolic acidosis of unknown etiology. Although there was poor but significant negative correlation with patients’ blood pH and bicarbonate levels when compared with plasma lactate levels reflecting the different etiologies, they showed a tendency to corroborate the observed lactic acidosis among the study patients. Furthermore, there were no significant differences with lactate levels obtained by the different sample handling conditions.

Several studies showed that lactate measurement has both diagnostic and prognostic value and that it should be measured within 3 h of presentation where lactate levels greater than 4 mmol/L are suggestive of sever sepsis [19]. Furthermore, in neutropenic patients, elevated lactate suggests that the patient is at risk of developing septic shock within the next 48 h [20]. Although not every patient with sepsis will have lactic acidosis (only 75% are thought to have elevated lactate levels [21]), lactate levels within the reference intervals are of prognostic value. Patients with circulating lactate levels within the upper part of the reference interval will exhibit poor outcomes when compared to those with lactate levels at the lower end [22]. Additionally, lactate measurement has been used to guide therapy where declining lactate is suggestive of appropriate intervention and of favorable outcome [23].

Achieving full compliance with recommended 15 min sample collection and handling time is unlikely to be met by most institutions and will thus impact lactate results. It would be helpful if manufacturers would consider, when developing guidelines on sample stability and on assays performance, constraints often seen in routine clinical practice.

To our knowledge, this study is unique in that it assessed sample stability among patient samples as well as normal subjects. This is an important concept when attempting to bridge the gap in pre-analytical processes.

There was no significance (P > 0.23 for all samples from normal subjects) when stored at either 30 min at 23 °C or at 30 min at 4 °C as compared to the manufacturer recommended (15 min at 23 °C). Similarly, there was no difference (P > 9.7) for patient samples when stored for 30 min at either 23 °C or at 4 °C when compared with the manufacturer recommendation. There was no difference for samples stored for 30 min at either 23 °C or at 4 °C from normal subjects (P > 0.24) or from patients (P > 0.97).
